# *Cell Proliferation *receives its highest ever Impact Factor of 4.936 and transitions to Open Access

**DOI:** 10.1111/cpr.12561

**Published:** 2019-01-29

**Authors:** Leah Webster

**Affiliations:** ^1^ Journals Publishing Manager John Wiley & Sons Oxford UK

Welcome to the first 2019 issue of *Cell Proliferation*. The journal has reached new heights and recently received its highest ever Impact Factor of 4.936 (© 2018 Clarivate Analytics). Over the past half‐century since *Cell Proliferation *first launched, the journal has witnessed continued developments within the field of cell biology and an explosion of published research. In recent years, we have received more submissions to the journal than ever before in its 50‐year history. It therefore seems a fitting time to look ahead to the next 50 years and take this opportunity to make some exciting changes to the journal.

We are constantly looking for ways to ensure that we continue to best serve authors’ needs—from maintaining the continued high quality of research published in the journal, to the speed and efficiency of our peer review and publication process. We are very pleased to announce that *Cell Proliferation *will move from being a subscription journal to an open access journal, effective from the 2019 volume.

Since 10th September 2018, all articles submitted and accepted for publication in *Cell Proliferation *have been published open access under a creative common licence and subject to an Article Processing Charge (APC). You can find details of the APC costs here: https://onlinelibrary.wiley.com/page/journal/13652184/article_publication_charges.html. You can find further details about *Cell Proliferation’s *open access licensing and copyright here: https://onlinelibrary.wiley.com/page/journal/13652184/open_access_license_and_copyright.html.

For authors, publishing open access creates more permissive use and sharing of their articles, which in turn helps to increase the dissemination of their research. *Cell Proliferation *has a global authorship base, many of whom are in regions where open access publication is accelerating. We also continue to see many institutions and global funders creating open access policies and mandates for their authors. As such, we would like to ensure that we are making it easier for authors to fulfil their funders’ mandates on open access publication. *Cell Proliferation *will join several other high impact journals in the Wiley Open Access portfolio (http://www.wileyopenaccess.com) and will benefit from the advantages that this confers. For readers, it will enable easier access to published research, giving greater visibility to both the authors and research published in *Cell Proliferation*.

From an editorial perspective, there will be no change to the journal’s current modus operandi. High‐quality, well‐conducted research is still imperative to *Cell Proliferation*, and all submissions will continue to undergo the same rigorous peer review process as they do currently. We hope that this change to an open access journal will encourage new authors and readers to *Cell Proliferation*. We are excited to see the journal embark on its new direction and hope that we can continue to count on you all, as readers and authors of *Cell Proliferation*, to submit your high‐quality research to the journal.

